# Nintedanib attenuates peritoneal fibrosis by inhibiting mesothelial‐to‐mesenchymal transition, inflammation and angiogenesis

**DOI:** 10.1111/jcmm.16518

**Published:** 2021-05-05

**Authors:** Feng Liu, Chao Yu, Huan Qin, Shenglei Zhang, Lu Fang, Yi Wang, Jun Wang, Binbin Cui, Susie Hu, Na Liu, Shougang Zhuang

**Affiliations:** ^1^ Department of Nephrology Shanghai East Hospital Tongji University School of Medicine Shanghai China; ^2^ Department of Medicine Rhode Island Hospital and Alpert Medical School Brown University Providence RI USA

**Keywords:** human peritoneal mesothelial cells, mesothelial‐to‐mesenchymal transition, nintedanib, peritoneal fibrosis, receptor tyrosine kinases, transforming growth factor β1

## Abstract

Nintedanib, an Food and Drug Administration (FDA) approved multiple tyrosine kinase inhibitor, exhibits an anti‐fibrotic effect in lung and kidneys. Its effect on peritoneal fibrosis remains unexplored. In this study, we found that nintedanib administration lessened chlorhexidine gluconate (CG)‐induced peritoneal fibrosis and reduced collagen I and fibronectin expression. This coincided with suppressed phosphorylation of platelet‐derived growth factor receptor, fibroblast growth factor receptors, vascular endothelial growth factor receptor and Src family kinase. Mechanistically, nintedanib inhibited injury‐induced mesothelial‐to‐mesenchymal transition (MMT), as demonstrated by decreased expression of α‐smooth muscle antigen and vimentin and preserved expression of E‐cadherin in the CG‐injured peritoneum and cultured human peritoneal mesothelial cells exposed to transforming growth factor‐β1. Nintedanib also suppressed expression of Snail and Twist, two transcription factors associated with MMT in vivo and in vitro. Moreover, nintedanib treatment inhibited expression of several cytokines/chemokines, including tumour necrosis factor‐α, interleukin‐1β and interleukin‐6, monocyte chemoattractant protein‐1 and prevented infiltration of macrophages to the injured peritoneum. Finally, nintedanib reduced CG‐induced peritoneal vascularization. These data suggest that nintedanib may attenuate peritoneal fibrosis by inhibiting MMT, inflammation, and angiogenesis and have therapeutic potential for the prevention and treatment of peritoneal fibrosis in patients on peritoneal dialysis.

## 1 INTRODUCTION

Peritoneal dialysis (PD) is an important renal replacement therapy for the end stage of renal disease (ESRD).^1^, ^2^ During this process, the peritoneal membrane is continuously exposed to hyperglycaemic and acidic dialysis solutions. Long‐term PD has been associated with progressive peritoneal membrane damage, submesothelial fibrosis, angiogenesis and vasculopathy, which eventually leads to ultrafiltration failure and discontinuation of PD.^1^, ^2^ Currently, effective clinical treatment for peritoneal fibrosis is limited.^2^, ^3^ As such, the development of an anti‐fibrotic therapy for targeting the key mechanisms in the peritoneal fibrogenesis is pressing.

Increasing evidence indicates that peritoneal mesothelial‐to‐mesenchymal transition (MMT) is an early mechanism of peritoneal fibrosis. MMT is featured by the disruption of intercellular junctions and conversion of mesothelial cells to a mesenchymal phenotype.^4^, ^5^ These transformed mesothelial cells can secrete profibrotic and angiogenetic cytokines that prompt cells to produce excessive amounts of extracellular matrix (ECM) components, inducing vascularization and mononuclear cell infiltration.^4^, ^5^ Similar to the epithelial‐mesenchymal transition (EMT) in other tissues, MMT results from growth factor—activation of multiple signalling pathways and transcription factors. Transforming growth factor‐β1 (TGF‐β1) has been shown to play a predominant role in peritoneal MMT and fibrosis. Other growth factors involved in MMT include platelet‐derived growth factor (PDGF), epidermal growth factor (EGF), fibroblast growth factor (FGF) and vascular growth factor (VEGF). These growth factors and their receptors are expressed in the injured peritoneum with receptor activation necessary for TGF‐β1 production. But pharmacologic targeting of one growth factor‐receptor pair may not be enough to block fibrosis since each pair may have a distinct role in the process of peritoneal fibrosis, such that application of a multi‐target receptor inhibitor might be an ideal therapy strategy to block multiple pathways in the process of peritoneal fibrosis.

Nintedanib is one such multi‐target receptor inhibitor that can simultaneously inhibit the phosphorylation of PDGFR, VEGFR, FGFR and the Src family kinases.^6^ It was originally developed as a drug for the treatment of various malignant tumours.^6^ In 2014, the FDA approved nintedanib as a treatment for idiopathic pulmonary fibrosis (IPF) because of its powerful anti‐fibrotic effect.^7^ Given the similar profibrotic mechanisms involved in other organs, it will be interesting to expand the anti‐fibrotic application of nintedanib beyond the lung.^8^ Recently, we found that administration of nintedanib inhibited the development and progression of renal fibrosis in a murine model of obstructive nephropathy.^9^ In cultured renal interstitial fibroblasts, nintedanib also suppressed TGF‐β1‐induced renal fibroblast activation and overproduction of ECM proteins.^9^ Since the initiation and development of peritoneal fibrosis are also a consequence of activation of multiple growth factor receptors, we speculated that nintedanib might also have a therapeutic effect in peritoneal fibrosis.

To test the hypothesis, we investigated the effect of nintedanib in a mouse model of peritoneal injury and fibrosis induced by chlorhexidine gluconate (CG). Our results showed that injury to the peritoneum induced phosphorylation of PDGFR, VEGFR, FGFR and Src and that treatment with nintedanib inhibited their phosphorylation and attenuated peritoneal MMT, inflammation and angiogenesis.

## 2 MATERIALS AND METHODS

### 2.1 Chemicals and antibodies

Antibodies to p‐PDGFR‐β, p‐VEGFR2, p‐Src, Src, p‐STAT3, STAT3, p‐Smad3, Smad3, TGF‐β1, p‐NF‐κBp65, p‐Akt, Akt and β‐Actin were purchased from Cell Signaling Technology. TGF‐β1 and antibodies to type I collagen and fibronectin were purchased from Santa Cruz Biotechnology. p‐FGFR1 antibody was purchased from Life Span Biosciences. NF‐κBp65 antibody was purchased from Prosci Inc. Antibodies to CD68, CD31, MMP‐2, TIMP‐2, E‐cadherin, vimentin, Snail, Twist, and MCP‐1, TNF‐α, IL‐1β and IL‐6 ELISA assay kits were purchased from Abcam Inc. Nintedanib was purchased from Cayman. α‐SMA antibody, CG, Cell Counting Kit‐8 (CCK‐8) proliferation assay kit and all other chemicals were purchased from Sigma.

### 2.2 Establishment of mouse peritoneal fibrosis models and nintedanib administration

The peritoneal fibrosis model was established in male C57/BL6 mice weighing 24‐28 g (Shanghai Super–B&K Laboratory Animal Corp. Ltd.) as described in our previous study.^10^ Briefly, peritoneal fibrosis in mice was generated by intraperitoneal injection of 0.1% CG (dissolved in 0.9% saline) every other day for 21 days. Control mice were injected with an equal volume of 0.9% saline. To examine the effect of nintedanib on the development of peritoneal fibrosis, nintedanib at 50 mg/kg was immediately given by gavage after CG injection and then administered daily. Nintedanib was dissolved in 50 µL of DMSO and then diluted in 100 µL of 0.9% saline. Mice were randomly divided into four groups with 6 mice in each group: (1) Sham: mice were administered with an equivalent amount of saline and DMSO; (2) Sham + nintedanib: mice were administered an equivalent amount of saline and 50 mg/kg nintedanib; (3) CG: mice were administered with 0.1% CG and an equivalent amount of DMSO; (4) CG + nintedanib: mice were given with 0.1% CG and 50 mg/kg nintedanib. On day 21 after CG injection, the whole parietal peritoneum apart from the injection point was harvested, with half of the specimen fixed in 10% formalin for histologic evaluation and the other half frozen in liquid nitrogen for ELISA and immunoblot analysis. All the experimental procedures on animals were approved by the Institutional Animal Care and Use Committee at Tongji University, Shanghai, China.

### 2.3 Cell culture and treatments

Human peritoneal mesothelial cells (HPMCs) (Jennio Biotechnology) were cultured in DMEM (Sigma‐Aldrich) containing 10% FBS, 1% penicillin and streptomycin in an atmosphere of 5% CO_2_ and 95% air at 37°C. To determine the effect of nintedanib on the mesothelial‐to‐mesenchymal transition (MMT**)** of HPMCs in response to TGF‐β1, cells were starved for 24 hours by incubation with DMEM containing 0.5% FBS and then stimulated with TGF‐β1 (5 ng/mL) for 48 hours in the presence or absence of nintedanib at a final concentration of 400 nmol/L. The dose of nintedanib was selected according to our and other studies.^9^, ^11^ To determine the effect of nintedanib on peritoneal mesothelial cell proliferation in response to growth factors, starved HPMCs were treated with PDGF‐BB (10 ng/mL) for 36 hours in the absence or presence of nintedanib at various concentrations (0, 50, 200 and 400 nmol/L). Cell proliferation was measured by the cell counting kit‐8 (CCK‐8) assay (Sigma‐Aldrich) according to the manufacturer's instruction.

### 2.4 Immunoblot analysis

Immunoblot analysis of peritoneum tissue samples and HPMCs was conducted as described previously.^12^ The densitometry analysis of immunoblot results was conducted using Image J software developed at the national institute of health. The quantification data are given as the ratio between the target protein and loading control.

### 2.5 Histochemical and immunofluorescent staining

Formalin‐fixed peritoneum was embedded in paraffin and prepared in 3‐μm‐thick sections. Immunohistochemical staining was conducted on the basis of the procedure described in our previous study.^12^ To evaluate peritoneal fibrosis, Masson trichrome staining was performed according to the protocol provided by the manufacture (Sigma‐Aldrich). The collagen tissue area (blue colour) was quantitatively measured using Image Pro‐Plus software (Media‐Cybernetics) by drawing a line around the perimeter of the positive staining area, and the average ratio to each microscopic field (×200) was calculated and graphed. CD31 and CD68 expression in peritoneum tissue were assessed by immunohistochemical staining.

### 2.6 ELISA analysis

To examine the expression of MCP‐1, TNF‐α, IL‐1β and IL‐6, mouse peritoneum was homogenized in an extraction buffer. The supernatant recovered after centrifugation was used for determination of these chemokine/cytokines by commercial Quantikine ELISA kits in accordance with the protocol specified by the manufacturer (AbcamInc). Total protein levels were determined using a bicinchoninic acid protein assay kit. The concentration of cytokines in the peritoneum was expressed as picograms per millilitre of the sample volume based on the manufacturer's instruction.

### 2.7 Statistical analysis

All the experiments were conducted at least three times. Data depicted in graphs represent the means ± SEM for each group. Inter‐group comparisons were made using one‐way analysis of variance (ANOVA). Multiple means were compared using Tukey's test. The differences between the two groups were determined by the Student's *t* test. Statistically significant differences between mean values were marked in each graph. *P* < .05 was considered a statistically significant difference between mean values. All the statistical analyses were conducted by SPSS 20.0.

## 3 RESULTS

### 3.1 Nintedanib attenuates CG‐induced peritoneal fibrosis in mice

**FIGURE 1:**
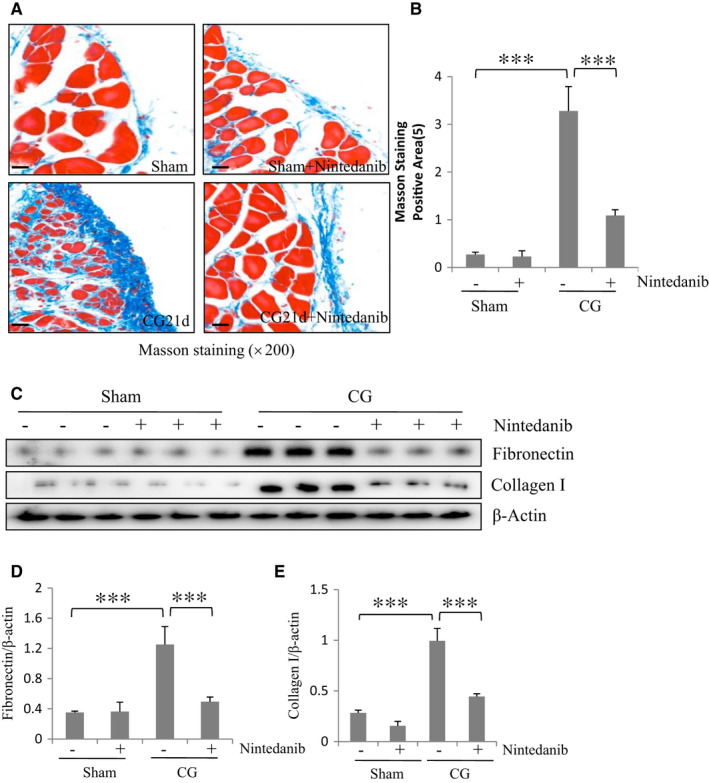
Nintedanib attenuates peritoneal fibrosis development in mice following CG injury. A, Photomicrographs illustrate Masson trichrome staining of the peritoneum with or without nintedanib treatment (×200). B, The graph shows the score of the Masson‐positive submesothelial area (blue) from 10 random fields (200×) (means ± SEM) (n = 6). C, The peritoneum was taken for immunoblot analysis of fibronectin, collagen I and β‐Actin. Representative immunoblots from 3 experiments are shown. Expression levels of fibronectin (D) and collagen I (E) were quantified by densitometry and normalized with β‐Actin. Data are means ± SEM (n = 6). ***P < .001. Scale bar:50 μm

Our previous studies showed that nintedanib at dose of 50 mg/kg attenuated renal fibrosis^9^; this dose was thus used to assess the effect of nintedanib on peritoneal fibrosis in this model. Nintedanib was given by oral administration immediately after CG injection and then daily for 21 days. As shown in Figure 1A,B, the thickness of the submesothelial zone and the area of collagen fibrils in CG‐injured mice with nintedanib administration were significantly smaller than in mice subjected to CG alone on Masson trichrome staining. To confirm the anti‐fibrotic effect of nintedanib, we further examined the impact of nintedanib on the expression of collagen I and fibronectin, two major ECM proteins deposited in the submesothelial compact zone of peritoneum, by immunoblot analysis and immunostaining. Immunoblot analysis demonstrated an increase in the expression of collagen I and fibronectin in the peritoneum after CG injection (Figure 1C‐E). Nintedanib significantly suppressed their expression. Similar results were also observed by immunochemical analysis (data not shown). As such, nintedanib may reduce interstitial expansion through suppression of ECM protein accumulation. These results suggest that nintedanib has a potential effect in preventing peritoneal fibrosis.

### 3.2 Nintedanib inhibits phosphorylation of multiple RTKs and Src in mice following CG injection

**FIGURE 2:**
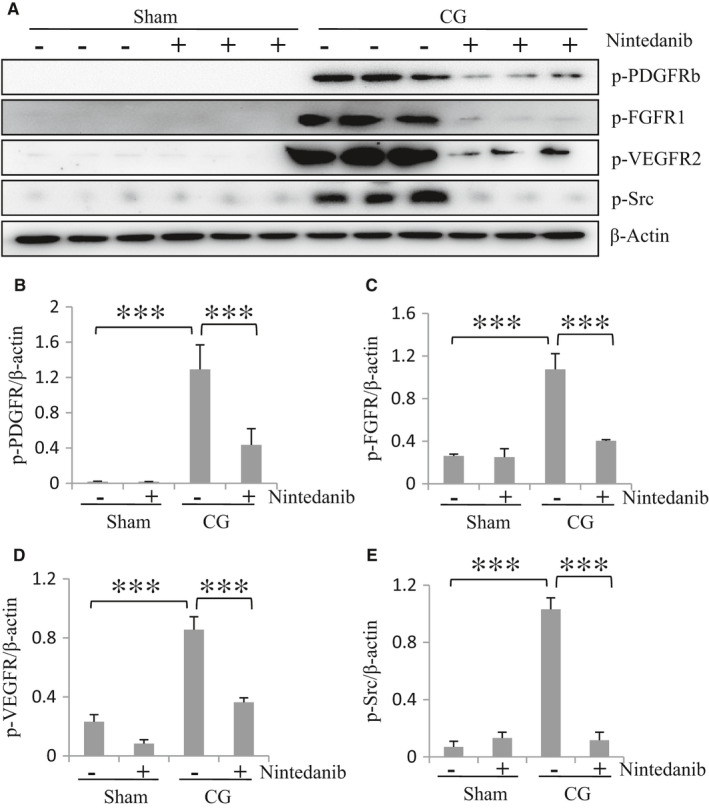
Nintedanib inhibits CG‐induced phosphorylation of multiple tyrosine kinases in the peritoneum of mice. (A) The peritoneum was taken for immunoblot analysis of phospho‐PDGFRβ (p‐PDGFRβ), phospho‐FGFR1 (p‐FGFR1), phospho‐VEGFR2 (p‐VEGFR2), phospho‐Src (p‐Src) and β‐Actin. Representative immunoblots from 3 experiments are shown. Expression levels of p‐PDGFRβ (B), p‐FGFR1 (C), p‐VEGFR2 (D) and p‐Src (E) were quantified by densitometry and normalized with β‐Actin. Data are means ± SEM (n = 6). ***P < .001

It has been documented that nintedanib is an inhibitor of PDGFR, VEGFR, FGFR and the Src family kinases.^6^ To demonstrate the specificity of nintedanib on the activation of these kinases in the peritoneum, we examined its effect on the phosphorylation of PDGFRβ, FGFR1, VEGFR2 and Src. CG injection for 21 days induced phosphorylation of PDGFRβ, FGFR1, VEGFR2 and Src whereas treatment with nintedanib largely reduced phosphorylation of each of them (Figure 2A‐E). Thus, we verified nintedanib as a potent inhibitor of these tyrosine kinases, which is consistent with our results observed previously in a murine model of UUO‐induced renal fibrosis.^9^

### 3.3 Nintedanib inhibits MMT in the peritoneum after CG injury

**FIGURE 3:**
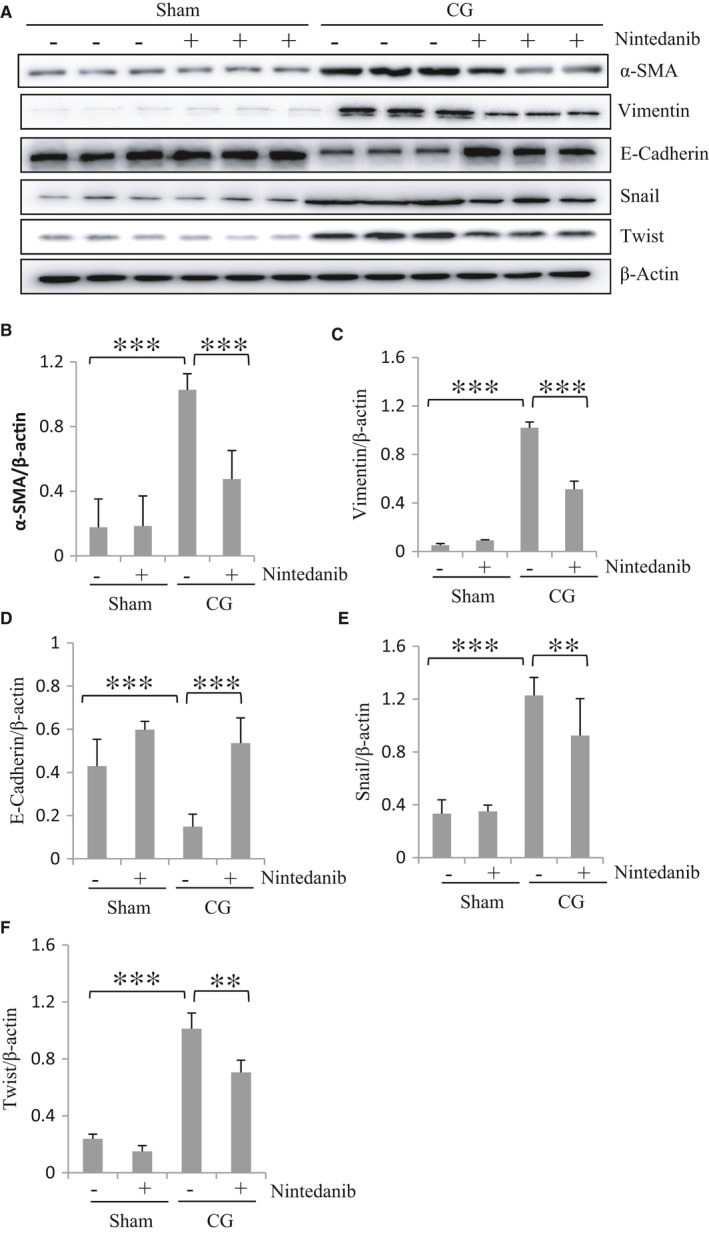
Nintedanib inhibits MMT in the peritoneum after CG injury. (A) The peritoneum was taken for immunoblot analysis of α‐SMA, vimentin, E‐cadherin, Snail, Twist and β‐Actin. Representative immunoblots from 3 experiments are shown. Expression levels of α‐SMA (B), Vimentin (C), E‐cadherin (D), Snail (E) and Twist (F) were quantified by densitometry and normalized with β‐Actin. Data are means ± SEM (n = 6). **P < .01; ***P < .001

MMT plays a primary role in the development of peritoneal fibrosis and functional decline of the peritoneal membrane as a dialysis membrane.^13^ Increased expression of α‐SMA and vimentin, and decreased expression of E‐Cadherin are the major feature of MMT, driven by the activation of transcriptional factors like Snail and Twist.^14^, ^15^ We therefore examined the effect of nintedanib on the expression of these proteins in the peritoneum after CG injury by immunoblot analysis. As shown in Figure 3A‐D, CG injection resulted in decreased expression of E‐cadherin and increased expression of α‐SMA and vimentin; treatment with nintedanib largely preserved E‐cadherin expression but inhibited α‐SMA and vimentin expression. Similarly, nintedanib treatment suppressed expression of Snail and Twist (Figure 3A,E,F). These results illustrated that nintedanib is able effectively to inhibit MMT in CG‐injured peritoneum.

### 3.4 Nintedanib inhibits MMT and proliferation of cultured HPMCs

**FIGURE 4:**
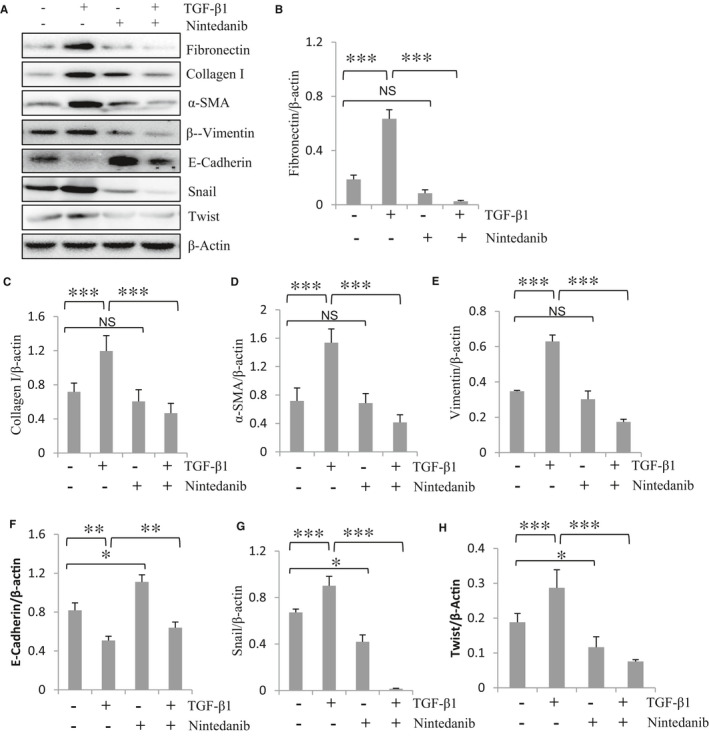
Nintedanib inhibits TGF‐β1‐induced MMT and expression of ECM proteins in cultured HPMCs. (A) Starved HPMCs were treated with TGF‐β1 in the presence or absence of nintedanib for 48 h and then harvested. Cell lysates were subjected to immunoblot analysis with antibodies against fibronectin, collagen I, α‐SMA, Vimentin, E‐cadherin, Snail, Twist and β‐Actin in HPMCs. Representative immunoblot from 3 experiments is shown. Expression levels of Fibronectin (B), Collagen I (C), α‐SMA (D), Vimentin (E), E‐cadherin (F), Snail (G) and Twist (H) were quantified by densitometry and normalized with β‐Actin. Data are means ± SEM (n = 6). **P < .01, *** *P* < .001, NS: *P* > .05

To verify the effect of nintedanib on MMT in vitro, cultured HPMCs were exposed to TGF‐β1, a potent inducer of MMT^16^ and then collected for immunoblot analysis of expression of MMT markers as well as Snail and Twist. As shown in Figure 4A‐H, exposure of HPMCs to TGF‐β1 resulted in increased expression of α‐SMA, vimentin, collagen I and fibronectin as well as Snail and Twist, whereas nintedanib treatment markedly inhibited their expression. In contrast, nintedanib treatment counteracted TGF‐β1‐induced E‐cadherin down‐regulation. These data support our in vivo observations that nintedanib is capable of suppressing MMT and resulting in production of ECM components.

Like nintedanib, treatment with gefitinib, a potent EGFR inhibitor, also attenuates MMT and peritoneal fibrosis.^12^ We sought to explore whether nintedanib would reduce TGF‐β1‐induced profibrotic responses in association with EGFR inhibition and whether nintedanib and gefitinib would have complementary effects. To that end, we examined the effect of nintedanib and/or gefitinib on the expression of α‐SMA, fibronectin and collagen I in HPMCs stimulated with TGF‐β1. As shown in Figure S1, treatment with either nintedanib (400 nmol/L) or gefitinib (10 nmol/L) partially reduced TGF‐β1‐induced expression of α‐SMA, fibronectin and collagen I whereas combined treatment with nintedanib and gefitinib led to a greater inhibition of these proteins. As expected, gefitinib was effective in blocking TGF‐β1‐induced EGFR phosphorylation. Interestingly, nintedanib treatment reduced EGFR phosphorylation to a significant degree and enhanced the inhibitory effect of gefitinib. These data indicate that nintedanib is capable of inhibiting EGFR activation and that combined treatment with gefitinib and nintedanib has a synergistic effect on the expression of profibrotic proteins.

Proliferation of HPMCs also contributes to the development of peritoneal fibrosis,^17^ and PDGF stimulates HPMC proliferation.^18^ Thus, we further explored the effect of nintedanib on HPMC proliferation. We treated HPMCs with PDGF (10 ng/mL) in the absence or presence of different concentrations of nintedanib and then determined cell proliferation by CCK8 assay and analysed PDGFR phosphorylation by immunoblot analysis. Figure S2A shows that nintedanib inhibited proliferation of HPMCs in a dose‐dependent manner, reaching the maximum effect at 400 nmol/L. In parallel, nintedanib dose‐dependently inhibited PDGF‐induced PDGFR phosphorylation with complete blockade at 400 nmol/L (Figure S2B,C). To confirm the efficacy of nintedanib on the activation of other receptor tyrosine kinases, we also examined the effect of nintedanib on FGF‐stimulated FGFR phosphorylation in HPMCs and demonstrated a similar dose‐dependent inhibition on this receptor (Figure 2D,E). Collectively, these data indicate a potent inhibitory effect of nintedanib on cell proliferation and phosphorylation of PDGFR and FGFR in HPMCs.

### 3.5 Nintedanib reduces expression of TGF‐β1 in the peritoneum after CG injury and inhibits activation of TGF‐β signalling in cultured HPMCs

**FIGURE 5:**
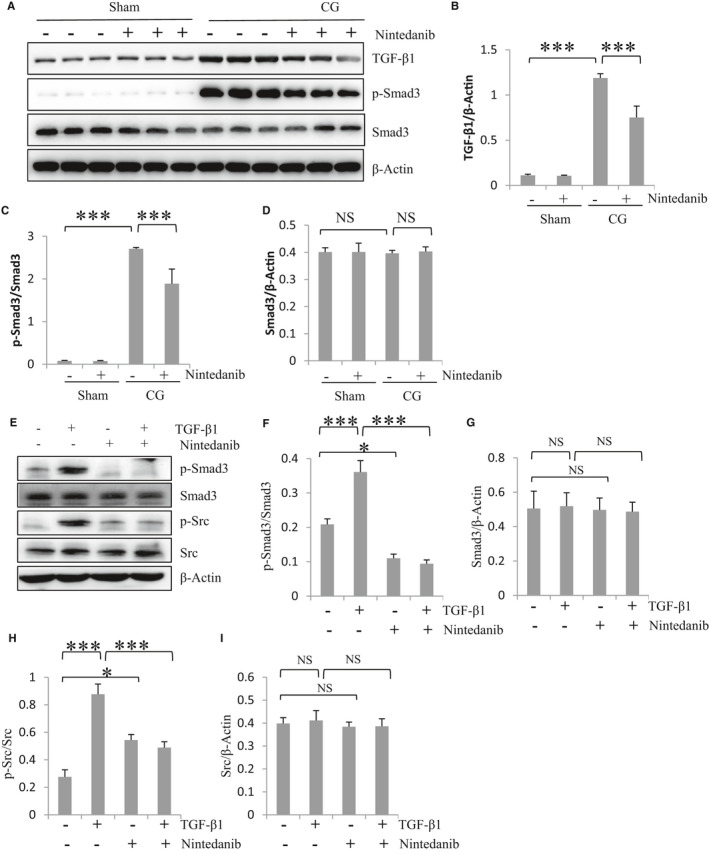
Nintedanib reduces expression of TGF‐β1 in the peritoneum after CG injury and inhibits activation of TGF‐β signalling in cultured HPMCs. (A) The peritoneum was taken for immunoblot analysis of TGF‐β1, phospho–Smad3(p‐Smad3), Smad3 and β‐Actin. Representative immunoblots from 3 experiments are shown. Expression levels of total TGF‐β1 (B) and Smad3 (D) were quantified by densitometry and normalized with β‐Actin. Expression levels of p‐Smad3 (C) were quantified by densitometry and normalized with total Smad3. (E) Immunoblot analysis shows the levels of p‐Smad3, Smad3, phospho‐Src (p‐Src), Src and β‐Actin in HPMCs after treatments by TGF‐β1 in the presence or absence of nintedanib. Expression levels of p‐Smad3 (F) and p‐Src (H) were quantified by densitometry and normalized with total Smad3 and Src, respectively. Expression levels of total Smad3 (G) and Src (I) were quantified by densitometry and normalized with β‐Actin. Data are means ± SEM (n = 6). *P < .05, **P < .01, ***P < .001, NS: *P* > .05

The TGF‐β signalling pathway plays a major role in MMT and the development of peritoneal fibrosis.^19^, ^20^ Thus, we further examined the effect of nintedanib on the expression of TGF‐β1 and activation of Smad3, a major signalling molecule in this pathway. In the animal model of peritoneal fibrosis, CG injury to the peritoneum resulted in increased expression of TGF‐β1 and phosphorylation (activation) of Smad3; nintedanib administration largely inhibited these responses (Figure 5A‐C). Notably, expression levels of total Smad3 remained the same in all groups (Figure 5A,D). In cultured HPMCs, TGF‐β1 treatment also increased phosphorylation of Smad3 and Src; presence of nintedanib reduced Smad3 and Src phosphorylation without significantly altering expression of their total protein levels (Figure 5E‐I). These data indicate that nintedanib can inhibit activation of TGF‐β1‐stimulated signalling pathway in vivo and in vitro.

### 3.6 Nintedanib suppresses production of multiple proinflammatory cytokines/chemokines and infiltration of macrophages in the peritoneum after CG injury

**FIGURE 6:**
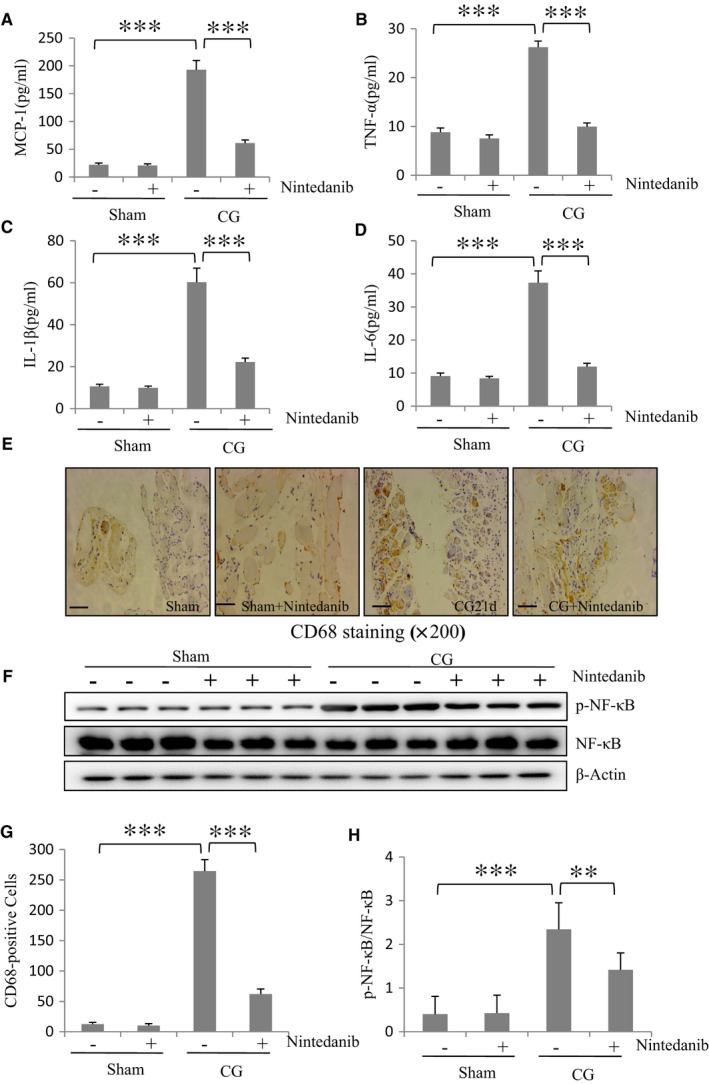
Nintedanib suppresses production of multiple proinflammatory cytokines/chemokines and infiltration of macrophages in the peritoneum after CG injury. Protein was extracted from the peritoneum of the mouse after CG injury with or without nintedanib administration and subjected to the ELISA assay for MCP‐1 (A), TNF‐α (B), IL‐1β (C) and IL‐6 (D). Photomicrographs illustrating immunohistochemistry staining of CD68‐positive cells in the kidney tissue treated with or without nintedanib for 21 d (E). The peritoneum was taken for immunoblot analysis of phospho‐NF‐κB (p‐NF‐κB), NF‐κB and β‐Actin as indicated (F). Representative immunoblots from 3 experiments are shown. The graph shows the percentage of immunohistochemistry‐positive area relative to the whole area from 10 random cortical fields (×200) (G). Expression levels of p‐NF‐κB/NF‐κB were quantified by densitometry as indicated (H). Data are represented as the means ± SEM (n = 6). **P < .01, ***P < .001. Scale bar: 50 μm

During peritoneal fibrosis, proinflammatory cytokines/chemokines are overproduced and inflammatory cells infiltrate the submesothelial compact zone.^21^ We thus examined whether nintedanib would be effective in suppressing expression in the peritoneum of major proinflammatory cytokines/chemokines and infiltration by macrophages of the peritoneum after CG injury. ELISA indicated that the expression of monocyte chemoattractant protein‐1 (MCP‐1), tumour necrosis factor‐α (TNF‐α), interleukin‐1β (IL‐1β) and interleukin‐6 (IL‐6) was elevated in the injured peritoneum; administration of nintedanib significantly suppressed their expression (Figure 6A‐D). Immunohistochemistry staining illustrated that the number of CD68‐positive macrophages was increased in the submesothelial layer of mouse peritoneum after CG injury; after nintedanib treatment, their numbers were reduced (Figure 6E,G). As NF‐κB is a major transcriptional factor that regulates expression of proinflammatory cytokines and chemoattractants in peritoneal fibrosis,^22^ we examined its phosphorylation and expression under the same experimental settings (Figure 6F,H). CG injury to the peritoneum induced NF‐κB phosphorylation, which was significantly suppressed by nintedanib. The expression of total NF‐κB was not affected by CG or nintedanib treatment. Collectively, nintedanib is also effective in inhibiting the inflammatory responses in the fibrotic peritoneum.

### 3.7 Nintedanib reduces angiogenesis in the peritoneum after CG injury

**FIGURE 7:**
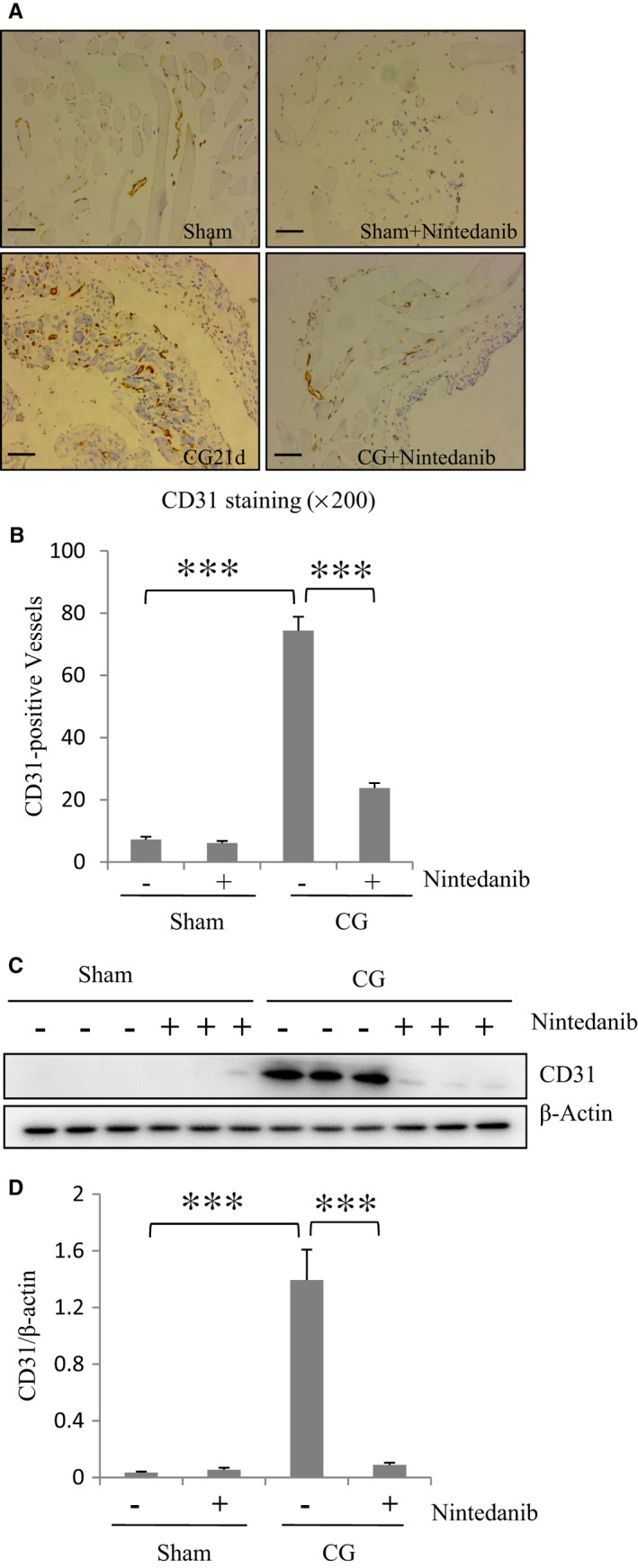
Nintedanib reduces angiogenesis in the peritoneum after CG injury. (A) Photomicrographs illustrating immunohistochemistry staining of CD31‐positive vessels in the kidney tissue treated with or without nintedanib for 21 days. (B) The graph shows the number of CD31‐positive vessels that was calculated from ten random fields (original magnification, ×200) of six mouse peritoneal samples. (C)The peritoneum was taken for immunoblot analysis of CD31 and β‐Actin. Representative immunoblots from 3 experiments are shown. (D) Expression levels of CD31 were quantified by densitometry and normalized with β‐Actin as indicated. Data are represented as the means ± SEM (n = 6). ***P < .001. Scale bar:50 μm

Angiogenesis, which occurs in the fibrotic submesothelial zone of the peritoneum during long‐term PD, is induced by VEGF released from injured mesothelial cells.^23^ Since nintedanib inhibits the interaction of VEGF with its receptors,^6^ we assumed that nintedanib might be able to interfere with angiogenesis in the peritoneum. To test this hypothesis, we examined the expression of endothelial cell marker CD31 in the peritoneum by both immunohistochemical staining and immunoblot analysis. Figure 7A‐B, showed that CG injury to the peritoneum led to an increase of CD31(+) vessels in the peritoneum while nintedanib significantly reduced the number of CD31(+) vessels. The results from immunoblot analysis also demonstrated that the nintedanib reduced the protein level of CD31 expressed in CG‐injured peritoneum (Figure 7C‐D). Therefore, nintedanib has a potent inhibitor effect on angiogenesis in the peritoneum injured by CG injection.

## 4 DISCUSSION

Peritoneal fibrosis is a major complication of long‐term PD and may eventually lead to failure of peritoneal ultrafiltration and termination of PD. Studies have demonstrated that activation of some receptor tyrosine kinase (RTKs) and Src superfamily kinases is involved in the initiation and development of peritoneal fibrosis.^19^ In this study, we found that nintedanib, a multiple tyrosine kinase inhibitor, was effective in inhibiting peritoneal fibrosis in a preclinical model of peritoneal fibrosis induced by CG. Moreover, we showed that administration of nintedanib inhibited MMT of human peritoneal mesothelial cells in vitro. These results indicate that nintedanib is a potential antagonist for preventing peritoneal fibrosis and suggest its potential as a novel treatment for peritoneal fibrosis.

The normal peritoneum is composed of a continuous monolayer of the mesothelial cells with a few fibroblasts, mast cells, macrophages and vessels in the submesothelial region.^1^ Daily exposure of the peritoneum during PD to dialysis fluid can cause acute and/or chronic injury of the peritoneal membrane.^8^ This process is coincident with the activation of several RTKs, including PDGFR, FGFR, VEGFR and Src, which have been shown to contribute to MMT and fibroblast activation.^24^ As a key regulator of angiogenesis, VEGFR can also induce an inflammatory response.^1^, ^19^ In this study, we found that administration of nintedanib inhibited deposition of ECM proteins, inflammation and angiogenesis, while suppressing the phosphorylation of all the RTKs and Src. This suggests that nintedanib can interfere with multiple pathological processes of peritoneal fibrosis by targeting these kinases. Since Src phosphorylation is initiated not only by the aforementioned three RTKs, but also other cellular membrane receptors, such as TGF‐β receptors and EGFR,^25^, ^26^ nintedanib may also inhibit peritoneal fibrosis initiated by other profibrotic factors. Supporting this concept, we demonstrated that nintedanib could inhibit TGF‐β1‐stimulated phosphorylation of EGFR, Smad3 and Src. Thus, because of its triple‐target tyrosine kinase inhibitory properties, nintedanib would have a more potent anti‐fibrotic effect than selective inhibitors of individual RTKs. In addition, we found that while either nintedanib or gefitinib, an EGFR inhibitor, can inhibit TGF‐β1‐stimulated expression of α‐SMA, fibronectin and type I collagen in HPMCs, their combined administration resulted in a greater inhibitory effect in cultured HPMCs. This suggests that combined administration of nintedanib and gefitinib would be more effect at attenuating peritoneal fibrosis than either one individually. Further investigations are necessary to examine the efficacy of the anti‐fibrotic effect of combined application of nintedanib and gefitinib in animal models of peritoneal fibrosis.

Peritoneal mesothelial cells (PMCs) undergoing MMT play an essential role in the pathological changes of the peritoneal membrane leading to fibrosis.^13^ In response to diverse stimuli, such as hyperglycaemia, growth factors and/ or inflammatory factors, PMCs lose their mesothelial characteristics and gain the feature of mesenchymal cells,^19^ acquiring the ability to secrete many inflammatory cytokines and overproduce ECM proteins.^19^ During this process, the two major transcription factors driving MMT, Twist and Snail, are involved in the down‐regulation of E‐cadherin.^27^, ^28^ In the present study, we indeed found that CG injury induces the MMT of PMCs, coincident with increased expression of Snail and Twist, while nintedanib inhibited their expression and preserved expression of E‐cadherin. In addition, we found that nintedanib treatment dose‐dependently inhibited PDGF‐induced proliferation of HPMCs. Given that PMC proliferation also contributes to peritoneal fibrogenesis,^1^, ^17^ our data suggest that nintedanib may attenuate peritoneal fibrosis via a mechanism associated with suppression of both MMT and PMC proliferation.

Another mechanism of nintedanib‐elicited attenuation of peritoneal fibrosis may be related to inhibition of inflammation. Infiltration by monocytes and macrophages and overproduction of proinflammatory cytokines have been shown to accelerate peritoneal fibrosis.^29^ In this study, we observed a significant elevation of proinflammatory cytokines/chemokines and infiltration of CD68‐positive macrophages to the injured peritoneum following CG injury, while the administration of nintedanib inhibited all these responses. Additionally, nintedanib treatment inhibited the phosphorylation of NF‐κB, a key transcription factor associated with the production of many proinflammatory cytokines. Thus, nintedanib exhibited a powerful anti‐inflammatory effect in our animal model of peritoneal fibrosis. These results are consistent with nintedanib‐induced suppression of inflammation in animal models of IPF and renal fibrosis.^9^, ^30^ Nintedanib may protect against the inflammatory response through simultaneous inhibition multiple RTKs and Src and subsequent activation of their downstream signalling pathways, such as NF‐κB.

Inhibition of angiogenesis may also be a mechanism by which nintedanib alleviates peritoneal fibrosis.^23^ Angiogenesis, an important pathological process in peritoneal fibrosis, can be induced by long‐term exposure to peritoneal dialysates with high‐dose glucose.^23^ It has been reported that VEGF could stimulate the formation of new capillaries, leading to angiogenesis and peritoneal fibrosis.^31^, ^32^ In this study, we found that nintedanib treatment effectively reduced the number of CD31‐positive cells and limited the thickness of peritoneum area in mice with CG injury. In addition to its effect on VEGFR, nintedanib may also inhibit peritoneal angiogenesis through targeting Src. This is supported by our previous observations that selective inhibition of Src reduced the number of CD31‐positive cells in the injured peritoneum.^33^ Therefore, we suggest that nintedanib may inhibit peritoneal angiogenesis at least via targeting VEGFR and Src.

We cannot exclude the possibility that nintedanib may also suppress peritoneal fibrosis through inhibition of lymphangiogenesis. In this regard, it has been reported by Lin et al^34^ that nintedanib inhibited suture‐induced corneal lymphangiogenesis and inflammatory cell recruitment in vivo and in vitro. Increased expression of VEGF‐C induced by TGF‐β can trigger lymphangiogenesis during peritoneal fibrosis,^35^, ^36^ and increased peritoneal lymphatic vessels can increase absorption of dialysate with high‐dose glucose during PD treatment, thereby reducing the ultrafiltration of peritoneal membranes.^37^ Given that lymphangiogenesis contributes to inflammation, high solute transport and ultrafiltration failure in the peritoneum,^38^ it will be interesting to conduct experiments in the future to further explore whether nintedanib diminishes peritoneal fibrosis and improves ultrafiltration failure by inhibiting lymphangiogenesis.

Although nintedanib has been approved clinically for the treatment of pulmonary fibrosis, its clinical value has not yet been assessed in other fibrotic diseases. Recent preclinical studies have demonstrated a potent anti‐fibrotic effect of nintedanib in many organs, such as liver, kidney, heart and skin.^8^ The current study has further extended the anti‐fibrotic effect of nintedanib to peritoneal fibrosis. Clinical trials have provided evidence for the effectiveness, safety and tolerability of nintedanib in patients with IPF.^39^ The most common side effects of nintedanib are gastrointestinal, including diarrhoea, nausea, stomach pain, vomiting and decreased appetite.^40^ Nintedanib is metabolized mainly in the liver, and only a very small amount is excreted by the kidney, suggesting that the reduced renal function in patients on peritoneal dialysis may not lead to potentially toxic levels of nintedanib. This biological property of nintedanib makes it an ideal anti‐fibrotic agent for patients with end‐stage renal disease. On this basis, clinical trials are needed to test its suitability in patients on PD.

In conclusion, our present study provides the first preclinical evidence for the anti‐fibrotic effect of nintedanib in peritoneal fibrosis. The underlying mechanism of nintedanib‐elicited peritoneal protection may be related to its multiple‐target actions on RTKs and Src, along with their downstream signalling pathways leading to the MMT, ECM synthesis, inflammation and angiogenesis. Therefore, our results suggest a potential therapeutic application of nintedanib and other RTK antagonists in preventing and treating peritoneal fibrosis.

## CONFLICT OF INTEREST

The authors confirm that there are no conflicts of interest.

## AUTHOR CONTRIBUTIONS

**Feng Liu:** Conceptualization (equal); Data curation (equal); Formal analysis (equal); Funding acquisition (equal); Investigation (equal); Methodology (equal); Project administration (equal); Writing‐original draft (equal). **Chao Yu:** Investigation (equal). **Huan Qin:** Investigation (equal). **Shenglei Zhang:** Investigation (equal). **Lu Fang:** Investigation (equal). **Yi Wang:** Investigation (equal). **Jun Wang:** Investigation (equal). **Binbin Cui:** Investigation (equal). **Susie Hu:** Writing‐review & editing (equal). **Na Liu:** Investigation (equal). **Shougang Zhuang:** Conceptualization (supporting); Data curation (lead); Formal analysis (lead); Funding acquisition (supporting); Investigation (lead); Writing‐original draft (supporting).

## Supporting information

Fig S1‐S2
