# EXPRESSION OF CONCERN: Nintedanib Attenuates Peritoneal Fibrosis by Inhibiting Mesothelial‐to‐Mesenchymal Transition, Inflammation and Angiogenesis

**DOI:** 10.1111/jcmm.71217

**Published:** 2026-05-25

**EXPRESSION OF CONCERN**: F. Liu, C. Yu, H. Qin, S. Zhang, L. Fang, Y. Wang, J. Wang, B. Cui, S. Hu, N. Liu and S. Zhuang, “Nintedanib Attenuates Peritoneal Fibrosis by Inhibiting Mesothelial‐to‐Mesenchymal Transition, Inflammation and Angiogenesis,” *Journal of Cellular and Molecular Medicine* 25, no. 13 (2021): 6103–6114, https://doi.org/10.1111/jcmm.16518.

This Expression of Concern is for the above article, published online on 05 May 2021 in Wiley Online Library (wileyonlinelibrary.com), and has been issued by agreement between the journal Editor‐in‐Chief, Stefan N. Constantinescu; the Foundation for Cellular and Molecular Medicine; and John Wiley & Sons Ltd. The Expression of Concern has been agreed following a concern raised by a third party. An investigation identified a duplication between the first three p‐Smad3 bands and the first three p‐Src bands shown in Figure 5E. The authors acknowledged the duplication but were unable to provide the original blots. While the editors consider the conclusions of the article are not affected, the journal has decided to issue an Expression of Concern to inform and alert readers.

