# Supplementary material for: Nintedanib attenuates peritoneal fibrosis by inhibiting mesothelial‐to‐mesenchymal transition, inflammation and angiogenesis
Source: J Cell Mol Med. 2021 May 5;25(13):6103–14. doi: 10.1111/jcmm.16518 (PMC8256343; doi:10.1111/jcmm.16518)
Supplement: Supplementary file 1 — Fig S1‐S2 [file JCMM-25-6103-s001.docx]

**Supplement Figure 1. The synergistic effect of nintedanib and gefitinib in inhibiting TGF-β1-induced expression of α-SMA, fibronectin and collagen I in cultured HPMCs.** (A) Starved HPMCs were treated by TGF-β1 for 48 hours in the presence or absence of nintedanib and/or gefitinib and then collected for immunoblot analysis of α-SMA, fibronectin, collagen-I, phospho–EGFR(p-EGFR),EGFR and β-Actin. Expression levels of α-SMA (B), fibronectin (C) and collagen-I (D) were quantified by densitometry and normalized with β-Actin. Expression levels of p-EGFR (E) were quantified by densitometry and normalized with total EGFR. Expression levels of EGFR (F) were quantified by densitometry and normalized with β-Actin. Data are means± S.E.M. (n =6). * p < 0.05, **p<0.01, *** p < 0.001, NS: P>0.05.

**Supplement Figure 2. Nintedanib inhibits PDGF--BB induced cell proliferation and PDGFR phosphorylation, and FGF-induced phosphorylation of FGFR in cultured HPMCs.** Starved HPMCs were treated with different doses of nintedanib (0, 50, 200, 400 nM) in the presence or absence of PDGF-BB (10 ng/ml) (A-C) or FGF (10 ng/ml) (D-E) for 36 hours. **(**A) Cell proliferation was assessed by the CCK-8 assay, (B) Cells were collected for immunoblot analysis of phospho–PDGFRβ (p-PDGFRβ) and β-Actin, (C) Expression levels of p-PDGFRβ were quantified by densitometry and normalized with β-Actin. (D) Cells were collected for immunoblot analysis of phospho–FGFR1(p-FGFR1) and β-Actin, (E) Expression levels of p-FGFR1(E) were quantified by densitometry and normalized with β-Actin. Data are means± S.E.M. (n =6). * p < 0.05, **p<0.01, *** p < 0.001, NS: P>0.05.
